# Microbial Transglutaminase Is a Very Frequently Used Food Additive and Is a Potential Inducer of Autoimmune/Neurodegenerative Diseases

**DOI:** 10.3390/toxics9100233

**Published:** 2021-09-25

**Authors:** Aaron Lerner, Carina Benzvi

**Affiliations:** 1 Chaim Sheba Medical Center, The Zabludowicz Research Center for Autoimmune Diseases, Tel Hashomer 5262000, Israel; carina.ben.zvi@gmail.com; 2 Ariel University, Ariel 40700, Israel

**Keywords:** microbial transglutaminase, gluten, celiac disease, autoimmune disease, neurodegenerative disease, cross-linking, posttranslational modification of proteins, side effects, safety

## Abstract

Microbial transglutaminase (mTG) is a heavily used food additive and its industrial transamidated complexes usage is rising rapidly. It was classified as a processing aid and was granted the GRAS (generally recognized as safe) definition, thus escaping full and thorough toxic and safety evaluations. Despite the manufacturers claims, mTG or its cross-linked compounds are immunogenic, pathogenic, proinflammatory, allergenic and toxic, and pose a risk to public health. The enzyme is a member of the transglutaminase family and imitates the posttranslational modification of gluten, by the tissue transglutaminase, which is the autoantigen of celiac disease. The deamidated and transamidated gliadin peptides lose their tolerance and induce the gluten enteropathy. Microbial transglutaminase and its complexes increase intestinal permeability, suppresses enteric protective pathways, enhances microbial growth and gliadin peptide’s epithelial uptake and can transcytose intra-enterocytically to face the sub-epithelial immune cells. The present review updates on the potentially detrimental side effects of mTG, aiming to interest the scientific community, induce food regulatory authorities’ debates on its safety, and protect the public from the mTG unwanted effects.

## 1. Introduction

**Figure 1:**
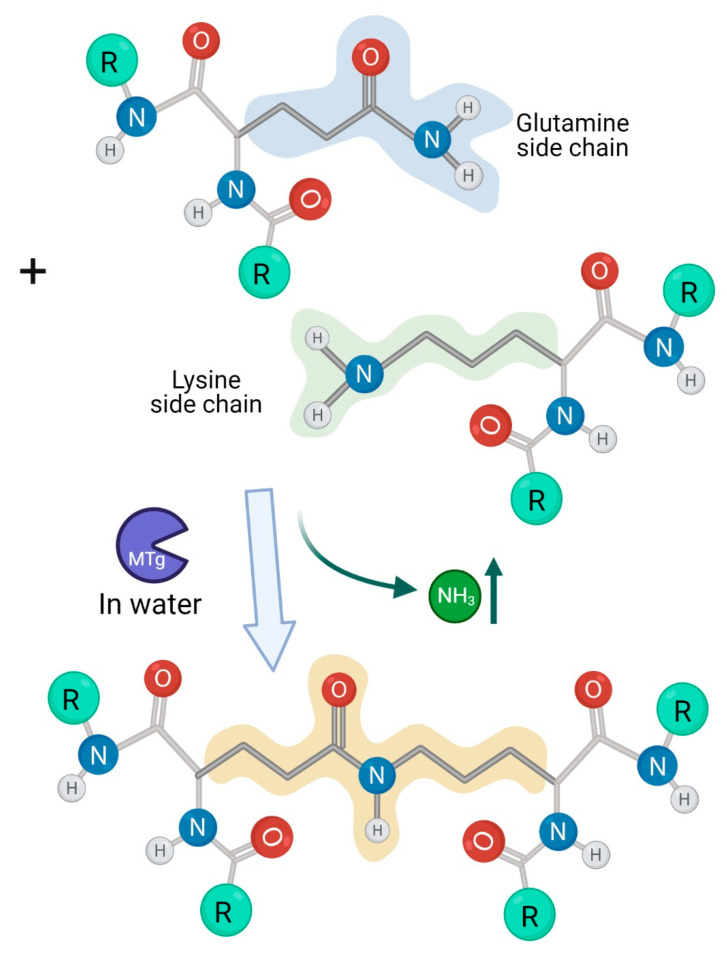
Microbial transglutaminase functions. When a glutamine residue and a lysine residue, on separated proteins, are incubated with mTG, a cross- linked covalent isopeptide bond is created releasing an NH3 molecule.

**Figure 2:**
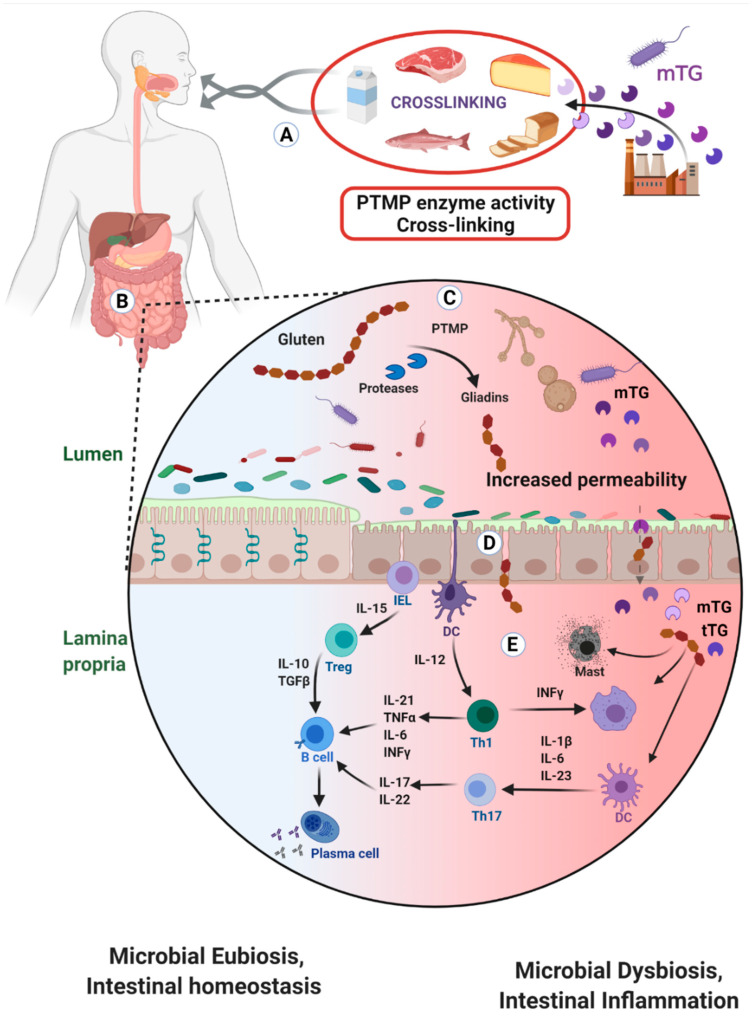
A schematic presentation of the mTG intestinal lumen sources, eco-events and immunogenic and pathogenic impacts. (**A**) Oral consumption of food products that were processed with mTG, such as meat, fish, dairy and bread. (**B**) mTG-peptides’ complexes reach the gut lumen. (**C**) Gliadins are rich in glutamine and lysine thus are a prime substrate for mTG cross-linkage, turning a naïve molecule to immunogenic one. More so, other mTG processed food products increase the enzyme luminal load for nutrients cross-linkage, and other organisms, such as yeast and fungi are an additional source of transglutaminase enzymes. The result is an increase in PTMP by the ability of mTG to deamidate or transamidate its substrates. Luminal digestive peptidases cannot break down these bonds, thus, inducing gut inflammation and damage to the intestinal epithelium. (**D**) mTG can potentially damage the lining mucus by breaking its stability and compromise tight junction functional integrity. Gluten increases intestinal permeability by binding to its epithelial CXCR3 receptor, resulting in zonulin release. Gliadin-mTG and other small peptide complexes might penetrate into the lamina propria through the open junctions or trans-enterocytically. (**E**) In the lamina propria gluten increases Th-17 activity, TLR4 signaling, NKG2P expression and neutrophil migration. mTG cross-linked complexes induce pro-inflammatory cytokines that drive T cells activation. Th1 secrete IFNγ and activates macrophages. Th17 secrete IL-17 and IL-22 which activate B cells. Two types of DC are present, the sub-epithelial one senses the lumen and regulates gut microbiota and another, mucosal one support Th1 immunity.

The transglutaminase secreted by bacteria is called microbial transglutaminase (mTG). Evolutionally, it is an important survival factor for prokaryotes like bacteria, fungi and actinomycetes. Many studies have been conducted to find microbial sources capable of secreting the enzyme due to its outstanding capacity to cross-link proteins or peptide [1,2,3]. Due to its avidity towards primary amine-containing substrates and its stringent specificity for high glutamine-containing proteins or peptides, the enzyme became a very practical tool to enzymatically form iso-peptide bonds between protein-protein and protein small molecule’s conjugates (Figure 1 and Figure 2). Microbial transglutaminase production, consumption and applications surged enormously in the last few decades [4,5,6,7,8,9]. In fact, its application has spread to processed food and textile industries, biomedical engineering, diagnostics and even to biomedical therapies [8]. Several recent reviews screened the potential health risks of mTG used in the food industries [3,9,10,11,12,13]. Microbial transglutaminase was recently suspected to be a new environmental factor in gluten depended conditions and neurodegenerative diseases [3,9,10,11,12,13,14,15,16]. The present review aim is to provide an update on the topic.

The transglutaminase family is a pleiotropic and a universal enzyme that is abundantly and ubiquitously expressed in living organisms, and the mTG is a member of the family. According to the classification, Transglutaminase (EC 2.3.2.13), i.e., protein-glutamine γ-glutamyltransferase, catalyzes the formation of an iso-peptide bond between the group of γ-carboxamides of glutamine residues (acyl residue donor) and the first-order ε-amine groups of different compounds, like proteins or peptides (acyl residue acceptors). Having the capacity of post translation modification of proteins, it can deamidate or cross-link substrates [17,18]. Its cross-linking ability gave the enzyme the nickname “meat glue” [8]. In this regard, mTG is heavily consumed in a plethora of processed food manufacturers. It improves meat appearance, texture, preservability and hardness, and improves texture and quality of liquid milk and dairy products. In the fish and marine industries, it enhances product hardness, improves protein film appearance and stability and even decreases caloric content. mTG is used in the sweet and confection industries to increase the product’s texture and elasticity. The enzyme is frequently used in bakeries, dairy, fisheries and seafood, confection, convenience, beverage, coffee, and even in oil industries [19,20,21,22]. Being a natural molecule, the mTG found its way even to the nutraceutical industries [23]. In addition to those wide applications, one of cost-effective advantages of mTG nutritional usage is the extension of shelf time in the supermarkets [24]. In reality and due to new applications, it represents one of the fastest-growing industrial areas. The number of patent applications filed on mTG is fast growing [3,16,21,25,26,27]. The global mTG market is anticipated to rise at a considerable rate in the next decade. Altogether, a maximum daily consumption of mTG ranges up to 15 mg. The estimated mTG doses to restructure 1 kg of food products is in the range of about 50–100 mg of the enzyme [3,9,12,28].

### 1.1. The Luminal Microbial Transglutaminase Sources in the Human Gut

Before embarking on the dark side of mTG usage in processed foods, the other luminal sources should be mentioned. Luminal mTG originates from extra-luminal as well as intra-luminal sources [3,11,12,28]. The extra-luminal load is represented by the amount of mTG consumed daily in processed food products. Evaluating mTG content in meat products, a substantial amount was depicted [29]. Intriguingly, the swallowed pathobionts, the probiotics, plants and vegetables are additional sources of mTG [3,11,12]. Microbial transglutaminase is used to increase the bioavailability of probiotics. mTG cross-linked soy protein isolate can improve the stomach passage of probiotics prior reaching the lower intestines [30]. No less important are the enteric microbiotic and dysbiotic cargos. In fact, the physiological microbiome, the pathogenic dysbiome, luminal yeasts, and fungi secrete the enzyme [3,11,12,31]. Interestingly, transglutaminase-like sequences were depicted in viruses, bacteriophages and mega phages, suggesting a common evolving from ancestral creatures [12]. Transglutaminase can be detected inside the gut lumen. Sloughed epithelial enterocytes or secreted transglutaminase can represent such sources for the tissue transglutaminase (tTG) [32]. In summary, the sources of gut lumen mTGs are diverse and mix together to perform their posttranslational modification of protein (PTMP) activities.

### 1.2. Microbial Transglutaminase-Gliadin Complexes Are Immunogenic in Celiac Disease

A major but not fully evaluated aspect is the potential detrimental effects of the mTG-gliadin complexes in celiac disease (CD). Despite having no similarity in their molecular weight, amino acid sequences, or factors that regulate their activities, both enzymes tTG and mTG share comparable functions. Both enzymes deamidate and cross-link suitable proteins/peptides, including gluten/gliadins [3,10,11,12,13,14,17,18,23]. In fact, due to their high glutamine and proline content, they are ideal substrates for both of the transglutaminases [3,12,28]. When applied together, tTG/mTG gliadin cross-linked complexes are formed, and new-epitopes appear inside and on the surface of the neo-complexes [3,12,28]. Since naïve gluten/gliadins are losing their immune tolerances, a new family of antibodies are generated. These are known as neo-epitope antibodies or neo-epitope autoantibodies and work against mTG or tTG, namely, mTG-neo and neo-tTG, respectively [3,13,28,33]. Both antibodies were reported in the serum of naive CD patients and are well known novel serological markers for CD diagnosis [33,34,35,36,37,38,39]. Multiple studies substantiated the immunogenicity of the mTG-gliadins complexes. By comparing, back-to-back, the most frequently used CD serological markers, the mTG-neo antibodies were as reliable as the others, approaching the sensitivity and specificity of the anti tTG [34,35,37]. In many reports mTG-neo antibody sensitivity and specificity was around 90–95%. Those antibodies nicely reflected the degree of the intestinal damage, as defined by March criteria, and had the advantage of early appearance during infancy [34,35,37]. Another aspect of mTG immunogenicity is when wheat is treated by the enzyme [12]. When applied on wheat, gluten or gliadins, several immune reactions can be detected. IgA anti-gliadin antibodies appear [40], and the reaction is age dependent [41]. There is a surge in intestinal interferon γ release and anti tTG and anti-endomysial antibodies appear [42]. Moreover, mTG treatment changed completely the electrophoretic pattern of maize and thousands of new bands were recognized by CD patients’ IgA [43]. Microbial transglutaminase treated wheat elicits immunoreactivity [44,45] and the products are recognized by gluten specific T cells [46]. Based on the above, mTG-neo antibodies were declared as new serological markers for CD diagnosis [33]. In summary, it is not the mTG itself, but rather its gliadin cross-linked complexes that are immunogenic, and the antibodies mounted against them reflect intestinal injury, appear early in life, and are diagnostic for the CD populations. Finally, there is an ongoing debate on mTG usage in bakeries. Some scientists advocate its therapeutic application [47,48,49,50] while others oppose it [3,9,10,11,12,13,17,18,34,35,37,51,52]. Well designed, double blind, cross-over CD patients’ challenges will clarify those contradictory opinions.

## 2. Microbial Transglutaminase Cross-Linked Complexes Are Pathogenic

The pathogenic pathways and mechanisms of the mTG and its transamidated complexes can be summarized as follows:

### 2.1. Trans-Enterocytic Transport of Gliadin and mTG

Gold tagging of gliadin and mTG allowed the following of the two molecules by electron microscopy. Both can be detected while trans-cytosed through early-late endosomes into the endoplasmic reticulum, to be deposited below the basolateral membrane of the enterocytic mono-layer. The author’s final conclusion was that: ”The strong localization of mTG at the basolateral membrane and the lamina propria may also indicate a potential antigenic interaction with cells of the immune system” [52]. Facing the sub-epithelial active immune systems, most probably, the mTG-neo antibodies are the outcome of this compartmental interaction. Notably, the mTG transamidated gliadins create stable covalent iso-peptide bonds known to be resistant to local peptidases, luminal bile acids and pH variations, thereby further challenging the local immune cells [3,10,12,13,28].

### 2.2. Compromised Tight Junction Functional Integrity

Multiple mechanisms can be suggested by which mTG itself or its gliadin cross-linked complexes can increase enteric permeability.

1. Zonulin, claudins, F-actin, occludins, myosin, F-cadherin, keratin and catenin present good substrates for mTG, since they contain acyl donors and acyl acceptors. Being essential for the tight junction performance, their mTG transamidation will open the enter-enterocytic gap [3,12,28];
2. Emulsifiers are disruptors of the gut tight junctions’ performances [9], and mTG has emulsifying activity [18,53,54];
3. Nanoparticles were designed to enhance intestinal permeability for drugs and nutrients. However, they have the potential to compromise human health [9,55,56,57,58]. On the other hand, mTG-designed neo-nanoparticles are increasingly used [59,60], hence, both add to increased gut permeability;
4. Pathogenic prokaryotes are powerful disruptors of human intestinal permeability [61,62]. Since mTG present a survival factor for the luminal microbes and since the mTG compromises some basic enteric physical and immune protective mechanisms, it might support luminal and mucosal pathobionts activities;
5. Gliadins and gluten are known to open the tight junction gap by stimulating zonulin release [61]. As an integral part of the mTG-gliadin neo-complex, the gluten/gliadin part of the complex can drive gut permeability. It should be noticed that this mechanism is not only shared between the CD patient, but also by their closed relative and to some degree the broader normal population [63,64];
6. Histones are mTG substrates and their cross-linking might result in free histone deprivation. Epigenetic is a major pathway in ADs development, including in CD evolvement [65,66,67];
7. Nutritional deficiency can induce a leaky gut. Glutamine and zinc deprivations are such an example [68,69,70].

Leaky gut could allow bacteria and its metabolome, toxins or many small molecules to ‘leak’ into the bloodstream. Even gliadins/gluten can be detected in CD blood or urine [15,69,71]. Since leaky gut/brain are associated, those factors might impact brain activity and be involved in neurodegenerative diseases and neurological/psychiatric presentations in ADs, including CD [72,73]. Indeed, processed food additives, cross-reactive nutrients, alpha enolase, tTG and potentially mTG are suspected to drive various human chronic disease, ADs and neurodegenerative included [9,14,74,75,76]. However, some questions deserve more studies. Since mTG cross-link its substrate, the differential part of the enzyme on tight junction integrity is not clear. One wonders how mTG performs when mixed with multiple nutrients during the meal and what the bioavailability of the enzyme inside the gut would be.

### 2.3. Enhances Enteric Epithelial Gliadins Uptake and Transportation

Apical-basal transfer of various gliadin peptides is assisted by secretory IgA and apical transferrin receptor when tTG is applied on epithelial cells [77]. More so, gliadins uptake is enhanced when tTG is applied on a cell line in vitro [12,28]. Since mTG functionally imitates it’s family member, the tTG, it is logical to assume that mTG can also facilitate mucosal gliadins uptake, thereby enhancing CD. However, the mTG effects on the blood- brain barrier is not known.

### 2.4. Suppression of Mechanical and Immunological Enteric Protective Barriers

An intact and functional mucus layer is a prime protective intestinal barrier in avoiding luminal detrimental factors and pathobionts to approach the enterocytes brush border. The mucus main structural compound is MUC2 mucin and due to its high glutamine and lysine content it represents an ideal substrate for tTG. In reality, the enzyme transamidates the MUC2 CysD2 domain, thus enhancing its protective function [78]. By adding the resistant isopeptide bond, mTG can perturbate mucin stability and fluidity resulting in detrimental attach of pathogenic luminal factors to the epithelial receptors. On the immunological level, mTG suppresses mucosal immune functions. *Streptococcus suis* mTG exerts antiphagocytic activity, thus suppressing a major immune protective mechanism [1,2,79,80].

### 2.5. Contributes to Luminal Microbiotic, Dysbiotic and Pathobiotic Proliferation

Being a survival factor for the microbes and a suppressor of gut immunity, mTG is a protective and growth factor for the Prokaryotes. When the *Streptoverticillium mobaraense* mTG gene was cloned into *Lactococcus lactis,* the bacterial mass increased significantly [81,82]. Newer bioengineered cloning of the mTG is successful in producing a higher yield and a more active form of the enzyme for a more cost-effective industrial application [83,84]. One wonders if a high mTG secreting bacteria will laterally transfer, by horizontal gene exchange, the mTG gene to the human microbiome, increasing its luminal yield and activity, thus perturbating luminal homeostasis [85].

### 2.6. Potential mTG-Gliadin Complexes Uptake and Presentation by Mucosal Dendritic Cells

The intestinal, intra or sub-epithelial dendritic cells with their elongations can sense, process and present luminal antigens [86,87,88]. It appears that monocyte-and macrophage-derived tTG are clearly involved in various inflammatory conditions [89]. The tTG derived macrophages and dendritic cells are capable to endocytose the enzyme [90,91], a process described by Stricker et al. [52] concerning the enterocyte’s transcytosis of the mTG and gliadins. In fact, the lumen is rich in mTG and digested gluten juxtaposed to the intestinal apical brush border. This new dendritic cell assisted transcytosis of tTG might represent a new port of entry for mTG and gliadins or cross-linked complexes to face the sub-epithelial immune cells [12].

## 3. Is mTG Active in the Human Gut Lumen?

As mentioned above, a substantial amount of mTG resides in the human enteric lumen [3,11,12,28,29]. There is no doubt that the mTG secreted by the luminal microbes is active. The question arises whether the contaminated food products or the mTG added to process the food is active inside the intestinal lumen. Several points were raised [92] and should be clarified [10].

- Are the mTG-gliadins cross-linked complexes destroyed in the stomach? As mentioned above, those covalent iso-peptide bonds are extremely resistant to the luminal proteases, reducing agents and detergents;
- Microbial transglutaminase is temperature dependent and is active up to 60° Celsius. In reality, many food products are not boiled before consumption or during processing, and some populations prefer eating raw meat. Just as a reminder, analyzing supermarket shelves’ meat and meat products, many were found to contain transglutaminase [29]. Intriguingly, mTG gliadin docked complexes turn more immunogenic when heated to 90° Celsius [10,52]. It is logical to speculate that during denaturation, epitopes are exteriorized and are exposed to the immune system. Regarding mTG activity and temperature, the newly identified cold Atlantic cod TG opens a new area of thermostable mTG application for boiled/heated/cooked food product’s manufacturing [93];
- Microbial transglutaminase is active at pH-4.0 and above. However, gastric physiology and pathophysiology show that upon eating or post-prandially, gastric acidity is neutralized. Large pediatric, adult and elderly people are chronically consuming acid suppressor medications, infants and elderly have higher gastric pH and alkaline reflux is not rare. Notably, the stomach pH is differentially distributed and some areas are less acidic [10]. In summary, it is suggested that active mTG can execute its functions in the duodenum, small and large bowel. The cross-linked complexes are created ex-vivo, while processing the food, they are stomach passage resistant and are immunogenic.

## 4. Should mTG Usage Be Labeled and Declared on Food Products?

For decades, the American regulatory authorities, the FDA, classified mTG in the GRAS category. They followed the manufacturers’ declarations on mTG being non-toxic, safe, non-allergenic, non-immunogenic and non-pathogenic for public health [3,12,28]. The topic of industrial enzyme production, usage and safety of genetically modified micro-organisms is the subject of intense debate, while continental and national discrepancies are wide [93,94,95,96,97,98,99,100,101]. Multiple issues are raised and the antibiotic resistance gene is of concern [85,94,95,96]. In view of continuous efforts to bioengineer more cost-efficient mTG for industrial applications [8,26,81,82,83,84,93] and in view of the all the detrimental effects of mTG and its trans-amidated complexes used for food processing (Figure 2), public health against the side effects of mTG should be a prime priority. The worldwide food and industrial safety regulatory authorities should reassess the updated observations; hence, consider the alleviation of the GRAS status and enforce the labelling of this heavily used processed food additive.

## 5. Should the Customers Be Warned for a Potential Health Risk of mTG Consumption?

The FDA’s GRAS category has evolved during the last decades [97,98] and attracted quite a lot of attention from scientists, regulators, policy-makers, professional and social media, and non-governmental organizations [99]. Critical opinions were expressed, including a recent one on the lack of a “master list of all GRAS chemicals used in food, nor did the FDA request the authority to do so from Congress” [100]. Another suggested inadequate “scientifically sound, rigorous, and transparent application of the GRAS concept” [101]. This reinforces the necessity for international assessments related to GRAS determinations [102]. Independent review in GRAS determinations is not obligatory, thus raising questions about the integrity of the evaluation [103]. The fundamental topic of conflict of interest between the FDA and the food manufacturers represent real concerns [103]. Above all, without knowing the substance’s features, activities, metabolism, physical-chemical characterization, or the optimal quantities in the food product, the “FDA can’t fulfill its statutory obligation for ensuring chemical safety of the U.S. food supply” [103]. On the other hand, despite taking a “hard look” on the GRAS FDA’s notices, Roberts et al. concluded that, in spite of this, the notes are “clearly defined, efficient, and cost-effective” [104]. Interestingly, several possible alternatives were suggested [105]. Safety concerns were raised in the pediatric field, even to the limits of urging “retesting all previously approved chemicals and labeling direct additives with limited or no toxicity data” [106]. The classification of the mTG in the GRAS category can present an example for the above-mentioned critics. The manufacturers’ declaration of the enzyme as being non-toxic, safe, non-allergenic, non-immunogenic and non-pathogenic for public health [3,12,28] does not match what is known in the literature. It appears that in the butcheries and bakeries mTG induces allergic reactions which are manifested by respiratory symptoms and categorized unflatteringly as “occupational allergens” [28,107,108]. The enzyme or its cross-linked complexes might be toxic, unsafe, immunogenic and pathogenic, being proinflammatory, increasing intestinal permeability and even auto-immunogenic [3,9,10,11,12,13,14,15,17,18,28,35,37,109,110]. Based on the widely criticized GRAS category, the detrimental effects of the mTG and its cross-linked complexes and the updated scientific literature, the national and international food regulatory authorities should reassess the “processing aid” classification of the enzyme. The mTG should be labeled as a food ingredient and meet standards that require maintaining public health.

## 6. Warnings for Use of Microbial Transglutaminase

Regulatory bodies, academic experts and social media opinion leaders are warning about mTG usage in the processed food industries. Multiple arguments have been raised against the unlabeled “processed aid” mTG. Following are some representative declarations: “The usage of transglutaminase as a food additive is permitted in some countries. However, its utilization has to be declared to ensure transparency for consumers” [29]. “Therefore, mTg can enhance the immunogenicity of gluten and should not be used in food products intended for consumption by CD patients” [46]. In fact, the worries and warnings on safe usage of the industrial enzyme exist in multiple publications [9,10,11,12,13,14,25,28,29,43,46,111,112,113,114]. Notably, in some European countries like Switzerland and Germany, or in Canada, the public was notified of potential public safety concerns, and recommended labeling the enzyme on the final product [115,116]. According to the EU Regulation No 1169/2011 dated 25 October 2011, reconstituted meat or fish products must include the word “formed” or “restructured” on the label. In contrast to European legislation, transglutaminase is not considered a processing aid that would be exempt from labeling by the FDA [117]. It is a dynamic process and the regulatory policies regarding food enzymes produced by engineered microbes and food additives is still evolving and should follow and respond to the new mTG biosynthetic methodologies and to its expanding application in the food chain [118,119].

## 7. Conclusions

Microbial transglutaminase is used as glue to cross-link proteins and other molecules during food processing. Nearly thirty years have passed since mTG appeared in the processed food industry. Its applications are continually expanding, as is its consumption [6,7,8,19,20,21,22,23,24,25,26]. As with many other food additives, there is often the dark side of it, but despite this, the enzyme is categorized as a processing aid and received the GRAS definition, thus escaping labeling and avoiding a more restricted toxic and safety evaluation. Microbial transglutaminase is a survival factor which acts by suppressing physical and immune protective mechanisms. It increases intestinal permeability and some of its transamidated products are immunogenic and pathogenic (Figure 2). Functionally imitating several human transglutaminases, mTG can be considered as a new potential environmental factor that might be implicated in several chronic human conditions (Table 1). Based on the above cited detrimental effects, the regulatory and food safety authorities should reconsider its status as a processing aid to a food ingredient that should be labeled and thoroughly evaluated for toxicity and public health safety. If discussed and applied, food additive policies, food labeling and regulatory product control will be substantially impacted. The proverb of Hippocrates coined in 400 BC: “let food be thy medicine”, does not apply to mTG cross-linked complexes. However, Benjamin Franklin’s declaration: “An ounce of prevention is worth a pound of cure.” applies much more accurately.

## Figures and Tables

**Table 1:** Chronic human diseases that are potentially associated with mTG.

Human Disease	References
Celiac disease	[3,9,10,11,12,13,17,18,28,33,34,35,37,38,39,41,42,43,44,46,51,52,112,113]
Dermatitis herpetiformis	[120]
Neurodegenerative diseases	[14,15,76,121]
Parkinson’s disease	[74]
Alzheimer’s disease	[121,122]
Huntington disease	[121,123]
Gluten ataxia	[124]
Allergic diseases	[107,108]
*Campylobacter jejuni* associated diseases	[125]

## Data Availability

No new data were created or analyzed in this study. Data sharing is not applicable to this article.

## Abbreviations

mTG- microbial transglutaminase, tTG- tissue transglutaminase, PTMP- posttranslational modification of proteins, CD- celiac disease, ADs-autoimmune diseases, GRAS- Generally Recognized as Safe.
